# Mesoscopic and microscopic imaging of sensory responses in the same animal

**DOI:** 10.1038/s41467-019-09082-4

**Published:** 2019-03-07

**Authors:** Davide Boido, Ravi L. Rungta, Bruno-Félix Osmanski, Morgane Roche, Tomokazu Tsurugizawa, Denis Le Bihan, Luisa Ciobanu, Serge Charpak

**Affiliations:** 1 INSERM U1128, Laboratory of Neurophysiology and New Microscopy, Université Paris Descartes, Paris, 75006 France; 2 NeuroSpin, Bât 145, Commissariat à l’Energie Atomique-Saclay Center, Gif-sur-Yvette, 91191 France

## Abstract

Imaging based on blood flow dynamics is widely used to study sensory processing. Here we investigated the extent to which local neuronal and capillary responses (two-photon microscopy) are correlated to mesoscopic responses detected with fast ultrasound (fUS) and BOLD-fMRI. Using a specialized chronic olfactory bulb preparation, we report that sequential imaging of the same mouse allows quantitative comparison of odour responses, imaged at both microscopic and mesoscopic scales. Under these conditions, functional hyperaemia occurred at the threshold of neuronal activation and fUS-CBV signals could be detected at the level of single voxels with activation maps varying according to blood velocity. Both neuronal and vascular responses increase non-linearly as a function of odour concentration, whereas both microscopic and mesoscopic vascular responses are linearly correlated to local neuronal calcium. These data establish strengths and limits of mesoscopic imaging techniques to report neural activity.

## Abstract (web summary)

Neuronal activity leads to a local increase in blood flow and volume, a process termed hyperaemia. Here, the authors employ multiple imaging approaches of neuronal and vascular activity at varying resolution to delineate the spatiotemporal dynamics of neurovascular coupling evoked by odours in the olfactory bulb.

## Introduction

Imaging techniques based on blood flow dynamics are commonly used to study sensory processing and higher cognitive function in the human brain. However, these techniques measure functional hyperaemia, a delayed increase of blow flow resulting from neurovascular coupling, which is made of a series of steps involving neurons, glial and vascular mural cells^1^. Several studies have suggested that in the brain, as initially shown in peripheral vessels, local activation of neurons generates a back-propagating signal from capillaries to upstream arterioles^2–6^, and this likely accounts for enlarging the vascular volume in the process of functional hyperaemia^4,7,8^. Therefore, functional hyperaemia has a larger “point spread function” than the neuronal responses driving it and we hypothesize that local neuronal responses trigger mesoscopic vascular responses over a fairly large area of the brain.

The olfactory bulb (OB) is an ideal model to test such a hypothesis due to its unique neuronal and vascular anatomy^9–11^ and its widespread use for investigating neurovascular coupling at both the microscopic and mesoscopic levels. Two-photon laser scanning microscopy (TPLSM) has revealed that in glomerular juxta-synaptic capillaries, odour triggers blood flow responses that are odorant specific, concentration dependent and correlated to local pre- and post-synaptic responses^12,13^. Vascular responses to odour have also been studied in the OB with two mesoscopic techniques, BOLD-fMRI and ultrafast ultrasound imaging (fUS). BOLD responses have been shown to be odour selective, located primarily in the glomerular layer, displaying adaptation and spatially overlapping with sensory inputs^14–21^. fUS, which measures changes in cerebral blood volume with higher temporal resolution^22–24^, was shown to detect vascular responses to odour within a couple of seconds in rats^25^. However, none of these studies have attempted to quantitatively compare the relationship between local synaptic activation and the resulting vascular signals, measured in the same animal, at both the capillary level with microscopic resolution, and at a more regional level with mesoscopic resolution. Comparison of vascular signals at these two different scales is important for several reasons: (1) fUS and BOLD-fMRI individual voxels contain multiple types and sizes of vessels, whose contribution to mesoscopic signals is unclear. (2) As recent findings have revealed that during functional hyperaemia, blood flow regulation involves several sites along the vascular arbour^4,26^, it is unknown whether quantitative mesoscopic and microscopic vascular signals similarly report local neuronal activation. (3) Correlating mesoscopic signals to the dynamics of locally measured capillary responses has the potential to improve the precision of mesoscale activation mapping.

Here, we developed an approach for sequential imaging of the same mouse with different imaging techniques under stable homoeostasis, i.e. in which signals can be reproducibly observed over weeks. In the OB of GCaMP6f expressing mice, we used TPLSM to image and analyze local neuronal Ca^2+^ signals and adjacent (juxta-synaptic) capillary blood flow changes within the same glomerulus in response to a wide range of odour concentrations. We compare these microscopic signals to fUS mesoscopic vascular signals acquired under the same experimental conditions. Finally, we extend this experimental protocol to BOLD-fMRI recordings in the same mice and quantitatively compare BOLD-fMRI and fUS signals.

## Results

### Neuronal and vascular responses in chronic mice

**Fig. 1:**
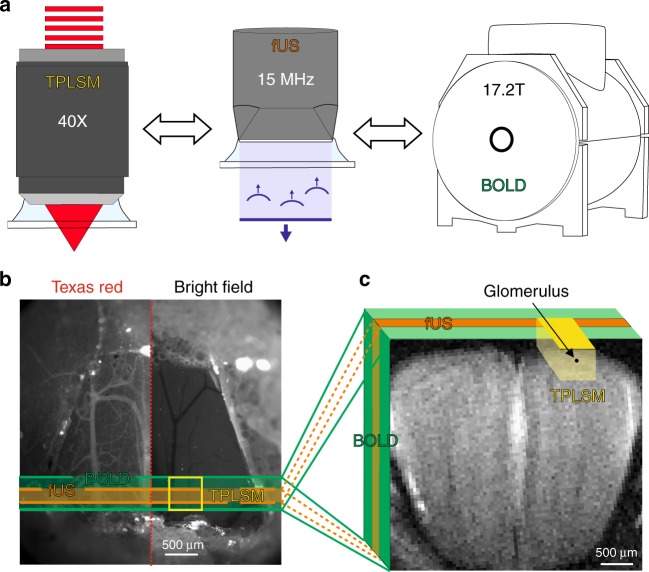
A chronic mouse preparation compatible for imaging with TPLSM, fUS and BOLD-fMRI. **a** Experimental design: imaging the mouse brain with TPLSM, fUS and 17.2T BOLD-fMRI. **b** Dorsal view of a mouse olfactory bulb (OB) imaged through a chronic PMP window. Left side: wide-field fluorescence microscopy with superficial vessels labelled with Texas Red Dextran. Right side: bright-field microscopy. The green stripe indicates the thickness (500 µm) of a slice imaged with BOLD-fMRI. The orange stripe indicates the thickness of the slice (200 µm) imaged with fUS. The yellow square indicates the field of view (500 × 500 µm) imaged with TPLSM in the same slice. **c** Schematic of the 3D volumes imaged with the three techniques and superimposed on an anatomical MR image acquired with a RARE sequence

In order to compare neuronal and vascular responses to odour across different techniques, we used a single preparation in which the materials were suitable for imaging with TPLSM, fUS, and BOLD-fMRI (Fig. 1a). Mice were implanted with a 125 µm thick polished polymethylpentene (PMP) cranial window over the dorsal OB (Fig. 1b, left), a material previously found acoustically transparent to ultrasounds^27,28^, and which we now report also allows TPLSM and BOLD-fMRI (T2* weighted FLASH) with no observed distortions. In order to be valid, the comparison between the techniques also required that responses to odour remain stable across days and weeks, allowing reproducible sequential imaging of the same animal. This was achieved in mice under sedation with an isoflurane/medetomidine specific protocol (see Methods). Importantly, the odour delivery was precisely calibrated before each experiment (see Methods). This overall approach enabled us to investigate how cellular responses to odour from individual dorsal glomeruli (TPLSM) quantitatively correlate to mesoscopic fUS and BOLD responses from the OB slice comprising the imaged glomeruli (Fig. 1b,c).

**Fig. 2:**
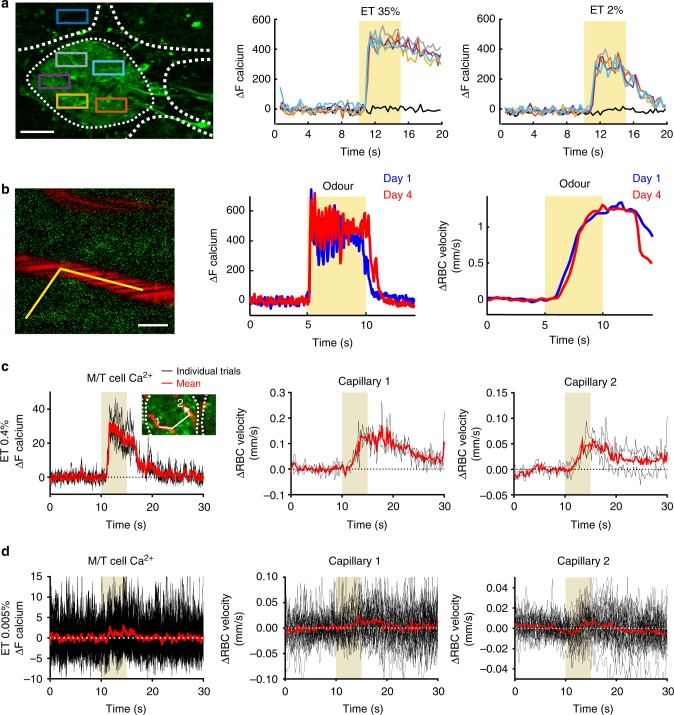
Characterization of vascular responses to odour in mice implanted with a chronic PMP window. Mice expressing GCaMP6f under the Thy1 promoter were imaged with TPLSM. **a** Odour causes a homogenous elevation of Ca^2+^ in mitral cell dendrites throughout the glomerulus. Left: Ca^2+^ was monitored in five ROIs located in the activated glomerulus and in one ROI located outside (black trace) (scale bar: 30 µm). Middle: ΔCa^2+^ dynamics and magnitude measured in all five ROIs comprised within the glomerulus were similar upon inhalation of ET (35%). Fluorescence did not change outside the glomerulus. Right: Ca^2+^ responses to 2% ET were smaller but similarly homogenous in the glomerulus. **b** Left: a broken line (in yellow) was used in the linescan acquisition mode to monitor Ca^2+^ in the neuropil and RBC velocity in a capillary (scale bar: 5 µm). Odour reproducibly causes a large Ca^2+^ increase the neuropil (middle), followed by an increase in RBC velocity (right). Both responses are very similar on day 1 and day 4 (averages of two inhalations). **c**, **d** Neuronal and RBC velocity responses remain coupled at 0.4% and 0.005% ET odour concentration. **c** Left: three single trials (black traces) and average (red trace) Ca^2+^ responses of the linescan segment between the two capillary segments (inset). Middle and right: corresponding RBC velocity responses in capillary 1 and 2, respectively. **d** Neuronal and RBC velocity responses persist at minimal odour stimulation threshold (ET 0.005%). Same as (**c**), except 30 individual trials (black) were averaged (red), as the single trial Ca^2+^ and RBC velocity responses were both hidden within the resting fluctuations. Baseline RBC velocities: **b** 0.35 mm/s; **c** and **d** 0.15 and 0.13 mm/s

All recordings were done in mice expressing GCaMP6f under the *Thy1* promoter (GP5.11 strain), which shows predominant expression in mitral/tufted (M/T) cells^29^, and within the dorsal area where M72 terminals typically project^30^. Figure 2a shows that the magnitude and dynamics of neuronal Ca^2+^ elevations within a single glomerulus were homogeneous between different regions of interest (ROIs), indicating that linescan acquisitions could be used to accurately represent glomerular Ca^2+^ dynamics, when simultaneously acquiring capillary red blood cell (RBC) velocity. Although neuronal and vascular responses can be unstable in acute mice, we found that in chronically prepared, sedated mice, the magnitude and temporal dynamics of both neuronal and single capillary RBC velocity responses were remarkably stable during ~ 90 min of recording sessions, as well as across different experimental sessions over days (Fig. 2b). The stability across days–weeks was also observed and quantified with micro- and mesoscopic imaging techniques (Supplementary Fig. 1). Finally, we tested how well vascular responses reported minimal levels of neuronal activation, since the existence of a threshold of neuronal activation, below which vascular responses are not triggered, remains an open and important question. Therefore, we investigated how local functional hyperaemia (i.e. ΔRBC velocity) reported neuronal activation at odour concentrations close to the threshold of dendritic Ca^2+^ elevations. Recordings were made within the most sensitive glomerulus to ethyl tiglate (ET), the area comprising this glomerulus being first screened in frame scan mode at low ET concentration (0.4%), and then isolated by reducing the concentration to 0.005–0.03% ET. At this concentration, Ca^2+^ signals were barely detectable in single trials, but evident upon averaging (Fig. 2d). At these low levels of stimulation, changes in RBC velocity were undetectable in single trials, however, upon sufficient averaging of the responses (26.4 ± 4.4 traces per mouse (mean ± SE)) the SNR improved, and as for Ca^2+^, RBC velocity increases became visible above the baseline and statistically significant (*p =* 0.008 for ΔCa^2+^, 0.008 for ΔRBC, Wilcoxon rank sum test, five mice, eight capillaries). The ΔCa^2+^ and ΔRBC minimal responses (0.005–0.03% ET) were approximately one-tenth of the 0.4% ET responses (ΔCa^2+^ minimal/ΔCa^2+^ 0.4% = 12.8 ± 4.8%; ΔRBC velocity/ΔRBC velocity 0.4% = 9.5 ± 3.5%, mean ± SEM). These results demonstrate that even minimal elevations in dendritic Ca^2+^ (at the threshold of detection with GCaMP6f) trigger functional hyperaemia.

**Fig. 3:**
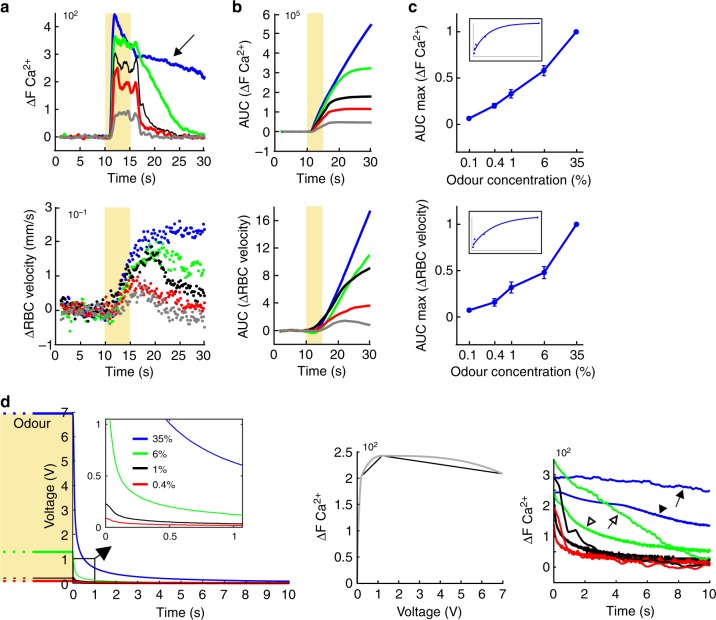
Concentration dependency of neuronal and capillary blood flow responses to odour. **a** Neuronal (top) and RBC velocity (bottom) responses to odour at increasing concentration. Each trace is an average of three trials. Note that at high odour concentration (arrow) the recovery of Ca^2+^ to baseline is delayed. Baseline RBC velocity: 0.25 mm/s. **b** Plots of the signal integral vs. time (area under the curve, AUC) for Ca^2+^ and RBC velocity responses. **c** Semi-log plots (AUC maxima) of summarized data for five mice. Values are normalized to the maximum value (35% odour). Data presented as mean ± SEM. Insets: exponential fits of the data with coefficients different from zero (CL > 95%). Baseline RBC velocities (mean ± SEM: 0.45 ± 0.19 mm/s). **d** At high odour concentration, enhanced neuronal responses are caused by delayed wash out across the 6 m delivery tubing. Left: time course of the odour measured in voltage (*V*) with a miniPID. The inset illustrates that when applied at 35%, the ET concentration 1 s after the offset remains important. Middle: plot of experimental ΔCa^2+^ values (black trace) taken at the end of the odour puff as a function of the miniPID voltage steps. A fitted curve with interpolated values is shown in grey. Right: traces of real and modelled ΔCa^2+^ decays as a function of time. Arrows point to real decays and arrowheads at model decays for 35 and 6% odours (blue and green traces, respectively). The persistence of odour at the end of the puff delivery accounts for a very significant proportion of the neuronal responses

We then established input–output curves over a range of odour concentrations (see Methods and Supplemental Information) applicable to all techniques. Although discrimination of odours occurs within a single sniff in the awake rodent^31–33^, we used 5 s odour stimulations spaced by 3.5–4 min, a protocol sufficient for triggering mesoscopic vascular signals while allowing time for full recovery of functional hyperaemia^34^, and minimal adaptation. This resulted in the ability to acquire on average 3–4 trials per odour concentration during a single experimental session. As expected, neuronal Ca^2+^ and capillary RBC velocity responses increased as a function of odour concentration (Fig. 3a–c). Baseline capillary RBC velocity responses had an average baseline velocity of 0.45 ± 0.19 mm/s and increased to 1.05 ± 0.40 mm/s upon inhalation of the highest odour concentration (35% ET, mean ± SEM, *n* = 5 mice). An additional effect was observed across multiple mice at high odour concentration (Fig. 3a): an increased duration of RBC velocity responses, which resulted from an enhanced activation of the glomerular network, illustrated by a shoulder in the Ca^2+^ signal. This enhancement resulted primarily from the prolonged time required for cleaning the high odour concentration in the 6 m tubing, necessary for delivering the odour in the magnet (see modelling in Fig. 3d as described in the Methods). These results demonstrate that in mice chronically implanted with a PMP window, juxta-synaptic capillary responses are tightly linked to the magnitude and duration of local neuronal activation over more than three orders of magnitude of odour concentration.

### fUS mesoscopic responses to odour

**Fig. 4:**
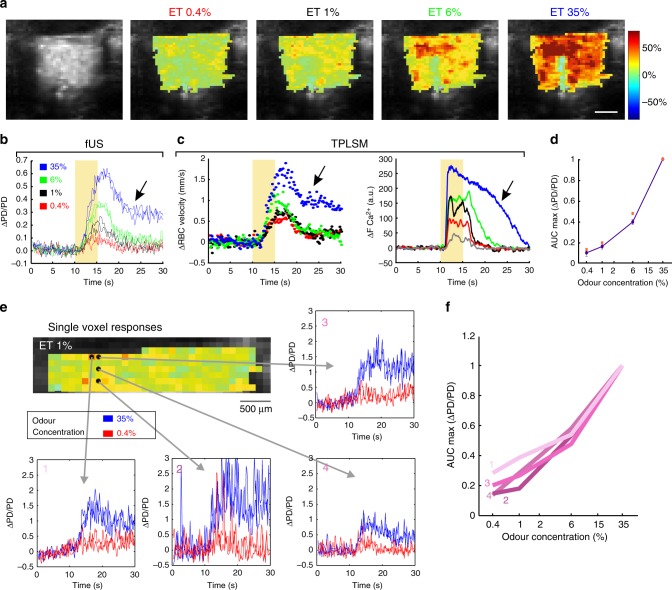
Comparison of mesoscopic (fUS Power Doppler) and microscopic (TPLSM) responses to odour. **a** Spatial distribution ΔPD/PD voxel values at increasing concentrations of odour (ET). Maps are superimposed onto a background image of the tissue signal (see Methods). Averages of three inhalations. Scale bar: 1 mm. **b** Time course of the global ΔPD/PD signal comprising all voxels (two trials are superimposed at each concentration). **c** RBC velocity (left) and neuronal ΔCa^2+^ (right) responses to odour in the same animal as **a**, **b**. Baseline RBC velocity: 1.25 mm/s. Traces represent mean of 2–3 trials. Note the similarity between the global fUS and single capillary RBC velocity response. Arrows (**b**, **c**) indicate prolonged neuronal and vascular activation at high odour concentration. **d** Semi-log plot of ΔPD/PD AUC maxima vs. odour concentration. Values are normalized to the maximum value (35% odour, *n* = 5 mice). Orange squares represent the mouse data from panel (**b**). **e** ΔPD/PD responses are detectable at the level of single voxels. Top left: dorsal region of **a** at 1% ET, showing upper seven voxel layers. For the four voxels, two trials are superimposed at two concentrations: 0.4% (red) and 35% (blue). **f** Semi-log plot of ΔPD/PD AUC maxima vs. odour concentration for each voxel (average of three inhalations, normalized to 35% ET). Data presented as mean ± SEM

In the olfactory bulb, specific synaptic activation of a single glomerulus triggers an increase of blood flow that spreads over several glomeruli, as functional hyperaemia is regulated by dilation of upstream arterioles and first orders capillaries^4^. Given the fact that vascular responses occur over a larger area than activated, we hypothesized that the vascular responses measured with fUS would encompass a spatial area larger than that of the glomerulus imaged with TPLSM. Therefore, we first used fUS to examine the odour concentration dependency of global functional hyperaemia measured within the entire OB slice that contained the glomerulus imaged with TPLSM. The spatial distribution of Power Doppler (PD) signals from all voxels within the slice, revealed that most of the OB was activated at high odour concentration (Fig. 4a). Averaging the ΔPD/PD signals from all voxels resulted in a global signal that increased with odour concentration (Fig. 4b–d), with the global vascular response within the slice being strikingly similar in dynamics to the single glomerulus capillary RBC velocity signal measured with TPLSM in the same mouse (Fig. 4b vs. c). Note that as for ΔRBC and ΔCa^2+^ (see previous paragraph), fUS responses at high concentration lasted longer than at lower concentration, a phenomenon due to prolonged odour stimulation and neuronal activation). Although each odour concentration was tested three times, the fUS sensitivity allowed detection of ΔPD/PD responses within single trials (Fig. 4b). In addition to extracting global signals, the high sensitivity of fUS also allows measurements of vascular responses at the level of single voxels (100 µm × 110 µm × ~ 200 µm)^28^. In response to odour, fUS signals could be readily detected at the level of single voxels with high repeatability from trial to trial, but with variability between the signals in different voxels (Fig. 4e), and similar dynamics in all layers, i.e. the glomerular layer (uppermost voxel layer), the voxel group containing the most responsive glomerulus (Supplementary Fig. 2) and the external plexiform and granular layers (Fig. 4f). Note that at high concentration, odour stimulation also induced a negative fUS signal in the medial region comprising high-velocity vessels (see next paragraph and Supplementary Fig. 3). Overall, these data suggest that the vascular response to odour has a wide spatial distribution within the OB, an observation we further investigated by building activation maps based on correlation with microscopic vascular responses.

### fUS activation maps

**Fig. 5:**
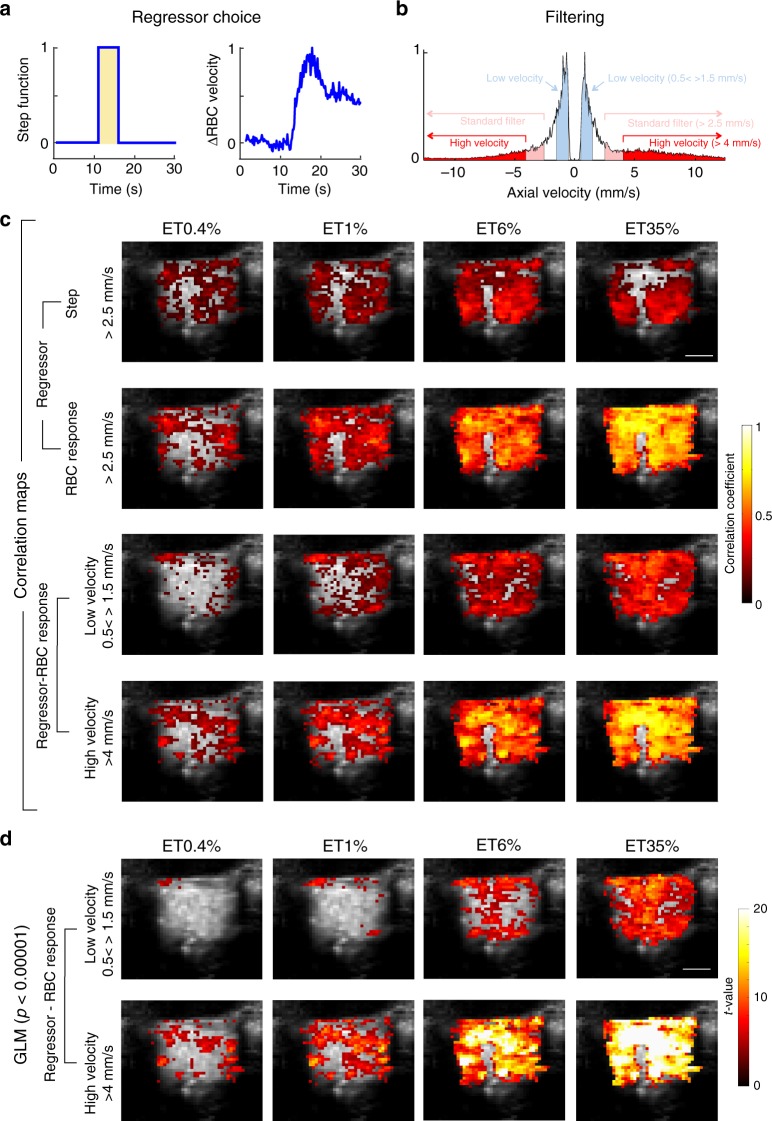
Mapping vascular activation with fUS. **a** Shows regressor used for building fUS activation maps: Left: step function; Right: capillary RBC velocity response (here at 35% ET). **b** Spectrum of Doppler frequencies converted to axial velocity (mm/s). Different filters were used for the analysis. Either a standard high-pass (HP) filter (reporting the CBV fraction flowing with an axial velocity > 2.5 mm), a higher HP filter reporting an axial velocity > 4 mm/s or a band-pass filter reporting an axial velocity between 0.5 and 1.5 mm/s). **c** Top two lines: comparison of the correlation maps obtained with the two regressors, using a standard HP filter. Using the RBC response as a regressor significantly improves the quality of the map, in particular in the dorsal OB. Bottom two lines: correlation maps for low axial velocities, i.e. comprising capillary responses differ from the large vessel maps (high axial velocities). **d** Activation maps of low and high axial velocity vessels using the general linear model with the RBC response as a regressor and a statistical threshold of (*p* < 0.0001)

OB maps of activated voxels were first constructed as maps of the normalized correlation coefficient (*r*)^24,28^ between each local ΔPD/PD voxel and either a 5 s step function (Fig. 5a, left) or the glomerulus single capillary ΔRBC response from the same mouse, measured with TPLSM for each odour concentration (Fig. 5a, right). Standard fUS analysis has used single value decomposition (SVD) filtering and an additional high-pass (HP) filter above 50 Hz, to remove tissue movement artifacts from the signal and report the CBV fraction flowing with an axial velocity > 2.5 mm/s^24,25,28^. Due to the high stability of our chronic preparation, we were able to decrease the cutoff filtering and obtain CBV maps flowing at both low (0.5–1.5 mm/s) and high (> 4 mm/s) velocity (Fig. 5b). Activation maps (*r* > 2*σ*, see the Methods section) revealed that at high concentration, most of the OB slice was indeed activated (Fig. 5c same animal as in Fig. 4). Using the measured microscopic vascular response (ΔRBC) as a regressor had two main advantages: (1) at high odour concentration, dorsal regions were strongly activated in contrast to what was extracted using a step function, the latter ignoring prolonged neuronal responses at high concentration. (2) At very low odour concentration, more activated voxels could be detected in the dorsal OB, from the glomerular layer to deeper layers. Note that at low odour concentration, the activation map was larger for high-velocity vessels.

We also constructed activation maps using statistical parametric mapping (SPM) and the general linear model, an approach commonly used for BOLD-fMRI analysis. Figure 5d illustrates that SPM activation maps with RBC velocity responses as the regressor (fUS frequency data was downsized from 500 to 10 Hz, *p* < 0.0001; t-contrast, no multiple comparisons adjustment) were very similar to the correlation maps (Fig. 5c). However, slightly fewer voxels were detected at the lowest odour concentration (0.4%), most likely due to differences in statistical threshold. Altogether, these data demonstrate that in chronically prepared mice, odour stimulation activates a vascular network that extends across and beyond the glomerular layer over a wide range of concentrations.

### BOLD-fMRI signals in mice imaged with TPLSM and fUS

**Fig. 6:**
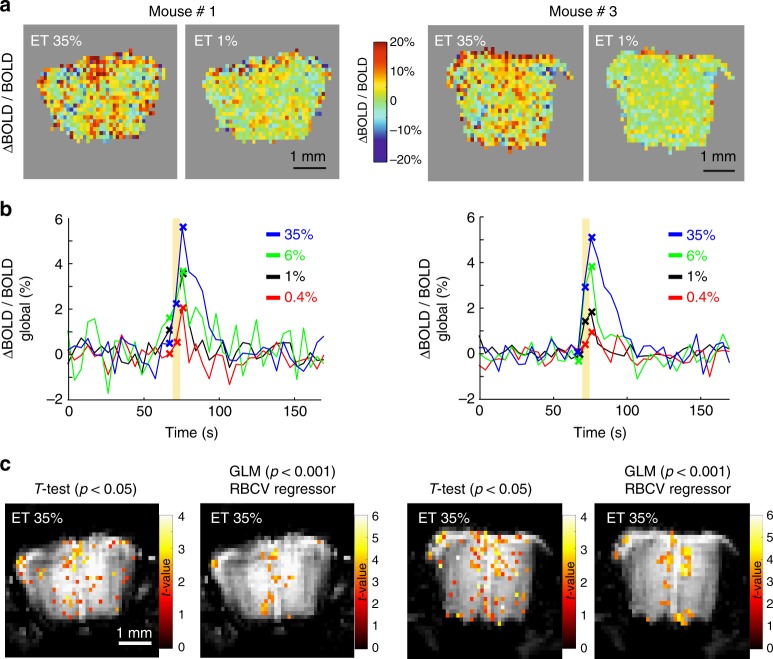
BOLD-fMRI imaging of odour responses in the same mice. Left: mouse #1 (shown in Figs 4 and 5). Right: mouse #3 (shown in Fig. 3). **a** ΔBOLD/BOLD signal voxel map within the OB mask (no threshold was applied). **b** Time course of the global ΔBOLD/BOLD signal (comprising all voxels). The response amplitude and duration increases with the odour concentration. Crosses indicate the individual data points before and after odour onset, showing that response occurs after the onset of the odour application. **c** BOLD-fMRI activation maps for the two mice using two statistical approaches; Left: *t*-test (*p* < 0.05); Right: the GLM (regressor: RBC velocity responses; a 2D smoothing was applied prior to regression, see Methods). In contrast to fUS activation maps, only few voxels are activated at 35% ET

As BOLD-fMRI is the leading method for human brain mapping, we tested whether BOLD-fMRI signals could be detected in the same mice, using the exact same protocol as for TPLSM and fUS, and three trials at each concentration. In contrast to the blood volume signals reported by fUS, the BOLD signal measures the effect of blood deoxyhemoglobin concentration changes and therefore depends on the complex interplay of oxygen consumption, blood volume and velocity changes that occur during functional hyperaemia^35^. Under our conditions at 17.2T, we found that in the mouse OB, echo planar imaging sequences induced large image distortions^20^, and therefore used a FLASH sequence to obtain high-quality images at the cost of lower temporal resolution. The global ΔBOLD/BOLD signal (from all voxels within the slice) increased with odour concentration (Fig. 6a, b), with dynamics similar to fUS and single capillary RBC velocity responses, in the same mouse (left, the same mouse as shown in Fig. 3; right, the same mouse as shown in Figs. 4 and 5). Using two different analysis approaches (either *t*-test or GLM with TPLSM RBC velocity responses as a regressor, see the Methods section), we were able to detect significantly activated voxels at 35% ET (Fig. 6c). However, despite the widespread vascular activation revealed by fUS at lower odour concentration (left, same mouse shown in Figs. 4 and  5), relatively few significant BOLD voxels were detected at ET 35%, even when using a less stringent statistical threshold (*p* < 0.001 for BOLD vs. *p* < 0.00001 for fUS; t-contrast, no multiple comparisons adjustment). This suggests that many activated voxels were lost in the noise, due to the low SNR of the BOLD signals upon averaging only three trials (5 s odour stimulation). For this reason, we limited BOLD mapping to high odour concentration (35% ET). To conclude, despite the low SNR of our stimulation approach (three trials, 5 s stimulation) that limited the reliability of the odour maps, global ΔBOLD/BOLD signals increased with odour concentration as seen with mesoscopic fUS signals.

### Quantitative comparisons of meso- and microscopic signals

**Fig. 7:**
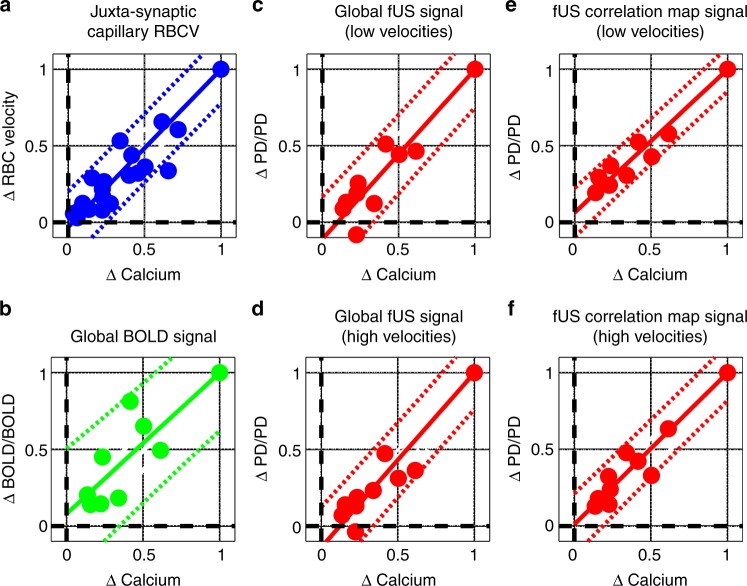
Correlation of glomerular Ca^2+^ with juxta-synaptic capillary and mesoscopic vascular signals. All vascular signals (both local and mesoscopic) are linearly related to glomerular Ca^2+^ increases over the range of odour concentrations tested. Integrals (AUC) of the signals are plotted on both axes, normalized to the response at the highest odour concentration (35% ET). **a** Simultaneous RBC velocity and postsynaptic Ca^2+^ responses at increasing odour concentration. **b** Global ΔBOLD/BOLD responses measured within the mask of the entire OB slice vs. ΔCa^2+^. **c**, **d** Global ΔfUS/fUS signal measured within the mask of the entire OB slice vs. ΔCa^2+^. The fUS signals are filtered to show responses from vessels with axial velocity between 0.5 and 1.5 mm/s (**c**) or > 4 mm/s (**d**). **e**, **f** ΔfUS/fUS responses comprising from all the voxels significantly correlated to the RBC response (see Fig. 5c as an example). Data points in each plot are responses to a range of odour concentrations from different mice (*n* = 5 mice in **a**, three mice in plots **b**–**f**). Dashed lines indicate 95% confidence level of the fit. Fits from individual mice are included in Supplementary Figure 4

The relationships between all three blood flow-related responses (ΔBOLD/BOLD global, ΔfUS/fUS and ΔRBC responses) with local dendritic ΔCa^2+^ responses could be well fit with linear functions (Fig. 7, coefficient of determination, *R*^2^_ADJ_ ≥ 0.98 for all plots but BOLD (≥ 0.92)). Linearity was not only a feature of the average measurements, but also applied to responses from individual mice (Supplementary Fig. 4). These data show that both the local and global vascular responses report the intensity of neuronal activation in the olfactory bulb over the range of odour concentrations tested. fUS input–output curves based on voxels correlated to RBC velocity responses had near zero intercepts (in contrast to global fUS signal curves, which included the additional effect of voxel recruitment with increasing concentration).

## Discussion

In this study, we compared microscopic and mesoscopic vascular responses to an odour stimulation and related these vascular responses to the activation of specific cells, the mitral and tufted cells which constitute the main output to the cortex. This comparison was made possible by developing a chronic window preparation and a sedation protocol compatible across techniques, thereby permitting the imaging of the different types of responses in the same mice. To compare the three techniques, odours were delivered with a stimulation duration (5 s) comprising the number of inhalations that are observed during natural sniffing bouts (about 15 = 3 sniff per second for 5 s)^36^. The odour concentrations ranged from 0.005 to 35% ET, 0.4% reflecting stimulation intensities commonly used to excite the olfactory system in awake animals^36^. This 5 s stimulation protocol combined with a 30 s acquisition time was ideal for TPLSM and fUS acquisitions. The 5 s odour application was associated with limited adaptation. The 30 s acquisition time was brief enough to avoid capillary photodamage and was also efficient for fUS imaging, as longer acquisitions required online processing and file saving times that limited the number of trials/imaging session. However, this short odour application and only three-trial averaging was not ideal for BOLD-fMRI mapping, due to the lower SNR and temporal resolution of our BOLD-fMRI protocol.

In contrast to the acute preparations in which the stability of neurovascular coupling can be variable, in our chronic mouse preparation (compatible across all techniques), we were able to follow neurovascular coupling over a large dynamic range, with coupling observed even at minimal stimulation. In addition to the development of a specialized window, this approach required an anaesthetic protocol that allowed stable recording for ~ 2 h. This was achieved by training the animals to our anaesthesia protocol. Although the physiological changes caused by this training are unclear, it generated a reproducible brain state during which the animal was sedated: it stayed still for ~ 2 h while remaining sensitive to a strong tail pinch. This approach allowed us to monitor neuronal vascular responses reproducibly over weeks. This adds information over previous cutting-edge techniques that simultaneously combined fibre-optic imaging with fMRI:^37–41^ TPLSM permits cellular resolution imaging of different cellular types or compartments (in our case, mitral cell dendritic tufts) and more importantly, monitoring of blood flow in individual capillaries, which was essential for our study. However, these improvements come at the cost of acquiring data sequentially rather than simultaneously.

We find that fUS is a very efficient technique for measuring mesoscopic vascular responses. Its high SNR and temporal resolution allows the generation of voxel-based correlation maps even at low-odour concentration. TPLSM and fUS imaging in the same mice allowed using an experimentally determined regressor (the capillary ΔRBC velocity response) for building activation maps, which resulted in significant improvements compared to standard fUS analysis approaches^25^. Additionally, we were able to establish CBV maps of vessels with low and high velocity by post processing with different band- or high-pass filters^42,43^. Interestingly, these maps suggest that at low-odour concentration, low-velocity responses were more localized to the glomerular layer where the highest density of capillaries is found. In contrast, high-velocity maps revealed more activated voxels in the deeper layers. In the absence of a precise map of the OB vascular architecture (in contrast to the vibrissa primary sensory cortex^44^), one can only suggest that deep layer responses result from either cortical feedback onto larger vessels in deep layers^21^ or the backpropagation of a vascular signal from juxta-synaptic capillaries to dilating arterioles in depth^4^. Furthermore, this arteriole dilation should also be expected to cause downstream non-specific signals in the glomerular layer, consistent with previous observations of capillary blood flow increases in some non-responding glomeruli^4,7^. Finally, a negative signal was reliably observed in the medial region, more evidently in the ΔPD maps because it overlapped to a high PD signal at rest (Supplementary Fig. 3). At this point, our data cannot discriminate whether the negativity results from an artery constriction or a steal effect.

In the most responsive glomerulus, local capillary responses are linearly correlated to calcium responses. This occurs over the entire range tested, and is exemplified by both the detection of responses at minimal stimulation and by the linear fit of the input/output curve which projects near the origin. This relationship shows that blood flow in the OB can be used as a quantitative marker of synaptic activation. Additionally, fUS and BOLD-fMRI global signals showed the same linear relationship with locally measured neuronal Ca^2+^ (note, neuronal calcium response was non-linearly related to odour concentration). The fact that the global response shows the same linear trend as the local vascular response is quite surprising, especially when considering the interplay of different mechanisms that are involved in the generation of increasing mesoscopic vascular responses in the OB: (1) the vascular signal back propagates from the most responsive glomerulus to the feeding arteriole^4^. (2) With increasing concentration, any odorant will progressively recruit non-specific glomeruli. (3) Although the glomerular layer contains the highest synaptic density, odour activates a network across the different layers of the OB, and recruits cortical feedback onto inner layers. (4) Even within a single voxel (fUS or BOLD), numerous vessels participate to the signal and their respective weight is unknown.

In other brain regions, the relationship between sensory evoked neuronal activity and mesoscopic responses has been widely investigated with laser Doppler, intrinsic optical signals and BOLD-fMRI and both linear and non-linear relationships have been reported (e.g., refs. ^45–48^, for review see refs. ^49,50^). One previous study investigated the relationship between sensory activation and fUS signals in the rat visual cortex, showing non-linearity in response to increasing visual contrast^51^, but the relationship to neuronal activity was not examined. Therefore, the degree to which the linearity observed here in the OB will extrapolate to other brain regions remains unclear.

The interpolation of the linear fits between M/T cell Ca^2+^ and either micro or mesoscopic vascular responses projected near the origin, suggesting that even minimal levels of neuronal activation could trigger elevations in blood flow. The high SNR of single capillary RBC velocity measurements with TPLSM allowed us to validate this hypothesis, as even when evoked Ca^2+^ responses and blood flow response were lost in the noise, at minimal stimulation, both signals appeared with ample averaging. It is important to stress that this absence of a mismatch between neuronal activation and functional hyperaemia is valid only if the most responsive glomerulus is imaged. Otherwise, blood flow increases can be detected in non-responding glomeruli^4,7^. Our data emphasize the importance of performing enough trial averaging to sufficiently increase the SNR for detection, whatever the imaging technique used. This is consistent with data from humans, which showed that massive averaging of BOLD-fMRI signals during a visual task results in the detection of many activated brain regions that were otherwise not detected^52^. In our experimental conditions, the smallest activation of neurons is accompanied by functional hyperaemia, suggesting that given sufficient averaging and SNR, mesoscopic imaging techniques could allow monitoring of brain activation over its full range.

## Methods

### Animal preparation

All animal care and experimentation was performed in accordance with the INSERM Animal Care and Use Committee guidelines (protocol numbers CEEA34.SC.122.12 and CEEA34.SC.123.12). Adult mice (*n* = 13, 2–12 months old, 20–35 g, both males and female, housed in 12-h light-dark cycle, fed ad libitum) were used in this study. *Thy1*-GCaMP6f (GP5.11) mice were obtained from Jackson laboratory. For chronic craniotomies^28^ mice were initially anesthetized with an intraperitoneal (IP) bolus of ketamine-xylazine (100 and 10 mg/kg body mass, respectively). An additional 10–20% of the same mixture was injected IP as necessary to maintain surgical plane of anaesthesia. During surgery, the mice breathed a mixture of air and oxygen; the body temperature was monitored using a rectal probe and maintained at 36 ± 0.5 °C by a feedback-controlled heating pad. The craniotomy was performed with a microdrill and care was taken not to apply pressure to the bone. The area was regularly flushed with cool aqueous buffer solution to avoid damage or heating of the underlying tissue. A piece of Polymethylpentene (PMP) (125 µm thick) was cut to the size of the window and sealed in place with Unifast 2 component dental cement (GC Dental Products, Tokyo, Japan) which was also used to form a head-cap in which a 3D-printed plastic head-bar was embedded. Mice were permitted to recover for at least 2 weeks before the experimental sessions began. For experiments, a sedation protocol in rats^53^ was modified for mice as follows. The mice were initially anesthetized with 3% isoflurane for 2 min, after which the mouse was attached to the head frame (while breathing 2% isoflurane) and a subcutaneous bolus of medetomidine (0.05 mg kg^−1^) was injected followed by a subcutaneous continuous perfusion of medetomidine (0.15 mg kg^−1^ h^−1^), that was maintained throughout the entire experiment. The isoflurane was gradually reduced by 0.5% every 10 min until at 40 min post-bolus injection when the isoflurane was completely removed. Recordings were not started earlier than 20 min from isoflurane cutoff. This protocol provided a reliable, stable sedation of the mice but only after two priming sessions during which we used the exact same anaesthetic protocol as for the subsequent recording sessions. These priming sessions were found compulsory to maintain animal stability for 90–120 min (measured by regular respiration that did not change upon inhalation of odours). During each experiment, breathing was monitored with a pneumogram transducer (Biopac Systems, Goleta, California, for fUS and TPLSM recordings; SA Instruments, Stony Brook, New York, for BOLD). The body temperature was monitored and maintained at 36.5 ± 0.5 °C using a rectal probe + heating pad; FHC (Bowdoin, ME) for fUS and TPLSM recordings, SA Instruments, Stony Brook, New York, for BOLD. Mice were supplemented with 29% O_2_ throughout the experiments.

### Odour stimulation

All the experiments performed with the different imaging techniques were conducted with no blinding and following the same stimulation protocol and materials (Teflon odour tubing and nose cone). A home built olfactometer based on the design of the Rinberg lab (https://www.janelia.org/open-science/olfactometer) and controlled by a custom Labview software was used to deliver the odours. The odour and exhaust lines were equilibrated at the start of each experiment. In order to ensure that the exact same odour concentrations were used across techniques, the odour concentration was calibrated before every experiment by measuring the odour puffs at all the odour concentrations tested with a photo-ionization detector that measures a calibrated voltage change/concentration (miniPID 200B, Aurora Scientific, Aurora, Canada). We additionally verified that the olfactometer was stable over the course of a day, by performing calibrations before and after experimental sessions. The odour concentration was determined by measuring the output at the end of the 6 m Teflon tubing. The final odour concentration values were calculated after considering the dilution from a supplemental O_2_ line that did not pass through the olfactometer. The 6 m tubing used across all techniques resulted in a smooth onset with a time of ~ 0.5 s to reach a plateau and a longer and concentration-dependent washout time (see Fig. 3). We interleaved the odour application of different concentrations during the experimental protocol for every imaging technique.

Note that fUS acquisition were shorter (30 s) than BOLD-fMRI acquisitions for two reasons: first, we wanted them to match TPLSM acquisitions and we have noted, through years of imaging, that repetitive linescan acquisitions lasting longer than 30 s could occasionally trigger non-physiological changes to capillaries (RBC stalls or changes in diameter). We have thus always stayed below the laser power and scanning duration that could generate this undesirable effect. Second, the time required for processing 30 s ultrasonic signals (after reception, e.g. for beamforming) required a few minutes. fUS recordings were thus limited to 30 s in order to keep the same interval between odour stimulations with all techniques.

### Odour wash out modelling

The prediction of the amount of calcium activation expected during odour wash out phase was calculated using a model based on interpolation (see Fig. 3). The first point of the predicted calcium signal was set as the last point of recorded calcium response at the end of the odour puff. Subsequent calcium points were inferred converting the recorded odour concentration decay at the end of the puff (measured by miniPID, in V) to the expected calcium signal. The expected calcium signal was calculated by interpolating the values of odour concentration vs. calcium on the dose–response curves. The dose–response curve for each mouse was made by fitting the average calcium response amplitudes at the tested odour concentrations with a cubic interpolation function (Matlab, interp1 function with ‘pchip’).

### BOLD acquisition

The MR acquisitions were performed on a horizontally oriented 17.2T small animal MRI scanner (Biospec, Bruker Biospin, Etlingen, Germany) using a custom built single loop surface coil (1 cm ID). For positioning the targeted field-of-view (FOV), multislice fast low-angle shot (FLASH) images were acquired. Good B0 homogeneity was ensured through automatic iterative FASTMAP methods (ParaVision 5.1), followed by a MAPSHIM correction^54,55^ in the region of interest. fMRI data were acquired using a 2D FLASH sequence with the following acquisition parameters: flip angle = 30°, field of view = 0.84 × 0.84 cm^2^, in plane resolution = 110 × 130 µm^2^, number of slices = 3, slice thickness = 500 µm, echo time = 6 ms, repetition time = 70 ms, number of repetitions = 50, acquisition time = 3 min and 44 s. Frames were acquired every 4.48 s. At the end of the fMRI session, high-resolution anatomical images of the same slices were acquired using a Rapid Acquisition with relaxation enhancement (RARE) pulse sequence: field of view = 0.64 × 0.64 cm^2^, in plane resolution = 50 × 50 µm^2^, echo time = 8 ms, repetition time = 2500 ms, RARE acceleration factor = 8, number of averages = 32, acquisition time = 21 min 20 s. For BOLD-fMRI recordings, a thin layer of Kwik-Cast (WPI, Sarasota, Florida) was put over the PMP window to avoid air susceptibility artifacts.

### BOLD data analyses

All analyses were performed with custom-made software developed in Matlab (MathWorks, Inc., Natick, Massachusetts) and SPM12 (Wellcome Trust Centre for Neuroimaging, http://www.fil.ion.ucl.ac.uk/spm/software/spm12/) fMRI suite. For analysis of the global signal, a mask of the OB was made by thresholding the averaged T2* signal across the time series. This analysis had minimal post processing (e.g. no spatial smoothing). Data realignment was performed using a 3-order polynomial interpolation and all trials at a given odour concentration were averaged voxel by voxel. The BOLD activation maps (percentage) show the average of four frames after odour onset subtracted from a baseline period of nine frames before odour delivery for each voxel. As in Fig. 3b, c, the AUC maxima (up to four frames (18 s) following the odour onset) was used to quantify BOLD responses. The peak AUC values at each odour concentration were normalized to the maximal odour concentration (35%) within each mouse to obtain the input/output curves. For analyses with SPM12 we aligned the frames to compensate for eventual movement/drift during the acquisition. For the *t*-test analyses, we compared the nine frames before odour delivery to a number of activated frames (expected from TPLSM data) dependent on the odour concentration (four frames for 35%, three for 6%, two for 1 and 0.4%). The statistical threshold was chosen in accordance to previous studies in the OB^19^. For the analysis using the general linear model (GLM), aligned data were 2D spatially smoothed using a gaussian filter with FWHM of twice the size of the voxels in the plane of the OB section. GLM regressors were made interpolating the RBC velocity responses to the same odour concentrations of the same mice. Because BOLD recordings lasted ~ 4 min while TPLSM scans 30 s, the points of the regressor before and after RBC velocity responses were set to zero. BOLD experiments were done on five animals (5/5 mice were also imaged and compared to fUS (Supplementary Figure 4), 3/5 mice were imaged with all three techniques to generate the curves in Fig. 7). In some regions where evident responses were observed with fUS, there were no significant BOLD-fMRI responses detected. However, BOLD was acquired with a much lower temporal resolution and our experimental design required testing several odour concentrations with the requirement to wait 3 min between trials, therefore limiting the total trial number per concentration. In order to accurately compare activation maps between the the techniques significantly, more trials at a single concentration would have been needed in order to increase the statistical power of the BOLD-fMRI acquisitions.

### FUS data acquisition and post processing

fUS imaging was performed as previously described^28^. Ultrasound-coupling gel was placed between the window and the linear ultrasound probe (15 MHz central frequency, 128 elements; Vermon; Tours, France). The transducer was connected to an ultrafast ultrasound scanner (AixplorerTM, SuperSonic Imagine; Aix en-Provence, France). Programming of custom transmit/receive ultrasound sequences was done in Matlab, using software-based architecture of the scanner. The mice bulbs were insonified with a succession of ultrasound plane waves and the backscattered echoes were recorded and beamformed to produce an echographic image for each transmission. To increase the SNR of each echographic image taken at 500 Hz, the echographic images were compounded by transmitting several tilted plane waves and adding their backscattered echoes. The compounded sequence resulted in enhanced echographic images, thereby increasing the sensitivity of the Doppler measurement without aliasing in the mouse brain. In this study, the ultrasound sequence consisted of transmitting eleven different tilted plane waves (−10 −8 −6 −4 −2 0 2 4 6 8 10° tilted angles) with a 5500 Hz pulse repetition frequency, giving a final frame rate of 500 Hz. As the backscattered signals from the mouse brain are composed of both tissue and blood signals, the following steps were performed to remove signals from the tissue. First, singular value decomposition^43^ was applied on the stack of the fUS images and the largest Eigenvalues were eliminated to filter out the slowest variations in the Power Doppler signal, which represented the tissue signal. Next, the backscattered signals were filtered with a fourth order Butterworth high-pass filter with the following cutoff frequencies: standard, 50 Hz high-pass; high-velocity, 80 Hz high-pass; low-velocity, 10–30 Hz band-pass. The Doppler signal of each spatial voxel was obtained by the incoherent temporal mean of the blood signal. The increase in Power Doppler signal was measured in individual voxels, which were 100 × 110 μm^2^ in plane size with a slice thickness of 200 μm. The background image upon which the ΔPD/PD signals are imposed in Fig. 4 (tissue signal) shows the first 40 components of the singular value decomposition filter^43^. These components account for the slowest variation of Power Doppler signal, representing the tissue.

### FUS data analyses

All analyses were performed with custom made software developed in Matlab. Post-processed data resulted in 500 Hz frame rate time series. OB masks were made based on the activation map at the highest odour concentration, which triggered activation within the entire OB coronal section we were recording from. The accuracy of the mask was verified by alignment on MRI anatomical image from the same mouse (+/−100 μm rostral/caudal). The top voxels of this mask were confirmed to accurately report the surface of the OB with a B-mode image that detects the interface between CSF and PMP. The peak AUC value was selected within 20 s after odour delivery, similar to the BOLD analysis of the same parameter. A 0.5 s moving average filter (Matlab ‘smooth’ function) was applied prior to peak finding because of the high frequency noise in the 500 Hz sampled fUS data. Similar to BOLD analyses, the signal intensity was averaged and then normalized to the maximal odour concentration for each mouse in order to calculate the input/output curves. For correlation analyses we calculated the Pearson correlation coefficient, *r*, between the local Power Doppler temporal signal computed from each spatial voxel of the fUS acquisition and either of two regressors: (1) a step function based on the odour application timing shifted by 1 s or (2) the time-course of the RBC velocity responses from the same mouse. The RBC velocity response was interpolated (cubic interpolation, ‘pchip’ option in Matlab ‘interp1’ function) to get the same sampling frequency as fUS. A threshold obtained from the average +2 SD of the correlation values against at least 50 voxels in the acoustic gel layer of each mouse was applied to the Pearson based correlation maps to visualize and select active voxels for further analyses^25^. Correlation-based fUS I/O (Fig. 7e, f) response to odours were computed averaging the ΔPD/PD value of the active (supra-threshold) voxels in each mouse/odour concentration. SPM12 analyses of fUS data was performed as follows. fUS time-course recordings of the average of three odour applications per concentration were single voxel under-sampled at 10 Hz with a cubic interpolation. Each frame was then converted to a NIfTI file. The absence of movement was previously assessed using a custom made Matlab script. No realignment or spatial smoothing was performed with SPM. GLM analyses used regressors from the same mouse/concentration RBC velocity responses. For the time reliability analyses, the ΔPD/PD responses of three odour applications for each tested mouse in the 2 days of recordings were averaged in time. The average response and the correspondent standard deviations were reported in a histogram plot. The coefficients of variation (CV) across days and trials were also computed and added to the plot containing the TPLSM data in Supplementary Figure 1. The corresponding location of two-photon glomerulus and fUS voxels was obtained by first matching the surface vasculature from two-photon images to the wide-field images of the craniotomy, then by measuring the distance between this location and the edge of the craniotomy. We then verified that the width of the fUS activation map was the same as the width of the craniotomy, and the glomerulus could be assigned to a voxel with a given error (see Supplementary Figure 2). We therefore decided to show the response time-course of both the central voxel and the six voxels centred on this voxel.

### TPLSM acquisition and data analysis

TPLSM imaging was performed using a custom-made acquisition system (National Instruments, Austin, Texas) and LabVIEW software (National Instruments, Austin, Texas). Femtosecond laser pulses were delivered by a Ti:sapphire laser (Mira by Coherent, Santa Clara, California, 120 fs pulse width, 76 MHz). An acoustic optical modulator (AA Optoelectronic, Orsay, France, MT110B50-A1.5-IR-Hk) was used to custom-modulate the laser power. Femtosecond pulses were targeted on the sample with galvanometric mirrors (Cambridge Technology, Bedford, Massachusetts). The excitation light was focused through a 40XW 0.8NA, or a 60XW 1.1NA objective (Olympus, Tokyo, Japan). GCaMP6f and Texas Red were excited at 920 nm. Emitted photons were separated based on their wavelength with a DCXR 560 (Chroma, Bellows Falls, Vermont) dichroic mirror. Red light was filtered with two E800SP, an E750SP and a GQ620/40 nm (Chroma), and reimaged onto a R6357 photomultiplier tube (Hamamatsu, Naka-ku, Japan). Green light was filtered with an E800SP2 and a GQ525/50 nm (Chroma) and reimaged onto a GaAsP photomultiplier tube (Hamamatsu). Texas Red dextran (70 kDa, Molecular Probes, ThermoFisher, Waltham, Massachusetts) was administered intravenously by retro-orbital injections. Following mapping of the ethyl tiglate activated region in frame scan mode and selection of the most sensitive glomerulus to the odour, broken linescan recordings were performed to record both RBC velocity and calcium transients in the neuropil, as previously described^28^. The power of individual segments of the broken line were modulated to optimize for differences in fluorescence of the plasma compared to GCaMP6f. Linescan acquisitions were repeated three times for each odour concentration, with the trials at different concentrations interleaved. Calcium data was interpolated (10 ms, cubic interpolation, ‘pchip’ option in Matlab ‘interp1’ function) and then trials at the same odour concentration were averaged. As with mesoscopic data, the peak AUC values were calculated from the average response at each odour concentration. Normalization was performed with respect to the highest odour concentration (35% ET). Time reliability assessment was performed similarly to fUS (see the ‘Statistical test' section for quantification). In addition to the five mice used to generate the input–output curve in Fig. 7, three mice were used to assess the threshold of functional hyperaemia and three mice were used to assess response reliability over days.

### Statistical tests

Sample sizes were chosen to be consistent with previous studies^20,46^. Exponential fits of the odour concentration vs. neuronal Ca^2+^ plots (insets Fig. 3) were performed with IGOR 4 (Wavemetrics Inc., Lake Oswego, Oregon). We tested the validity of every fit that properly converged to a minimum of the optimization function (least-square method) by verifying that every coefficient was statistically different from zero with a confidence level of 95% (value > 2*SD). Linear fits of the input/output distributions within each imaging technique were computed in Matlab and the degree-of-freedom adjusted coefficient of determination (*R*^2^_adj_) calculated for each plot (see Fig. 7). The significance of the minimal stimulation responses was assessed against the null hypothesis (mean response equal to 0) with non-parametric Wilcoxon rank sum test (further confirmed by 2-sample Kolmogorov–Smirnov test) (ΔCa^2+^ and ΔRBC velocity), as Ca^2+^ and RBC velocity responses did not pass a 1-sample Kolmogorov–Smirnov normality test evaluation. ΔCa^2+^ and ΔRBC responses were quantified as the mean signal during 2–6 s and 3–7 s following the onset of the odour application, respectively. The effect size and relative statistical power of the Wilcoxon rank sum test (G-Power, Dusseldorf University) was computed to evaluate, a posteriori, that our experimental size was adequate (effect size = 1.51 and 1.42, statistical power = 0.84 and 0.80 for ΔCa^2+^and for ΔRBC, *n* = 5 mice).

We evaluated the reliability over time using the CV (standard deviation/average) across trials and days using the average responses of calcium imaging, RBC velocity and fUS ΔPD/PD data. We computed the CV across the 2 days of recordings using average of the mean response from each day and their standard deviation, while the CV across trials was the mean of the CVs of the trials from each day (see Supplementary Figure 1).

### Reporting summary

Further information on experimental design is available in the Nature Research Reporting Summary linked to this article.

## Supplementary information

Supplementary Information

Reporting Summary

## Data Availability

The data that support the findings of this study and the code used for the analyses are available from the corresponding author on reasonable request.
