# Supplementary material for: Mesoscopic and microscopic imaging of sensory responses in the same animal
Source: Nat Commun. 2019 Mar 7;10:1110. doi: 10.1038/s41467-019-09082-4 (PMC6405955; doi:10.1038/s41467-019-09082-4)
Supplement: Supplementary file 1 — Supplementary Information [file 41467_2019_9082_MOESM1_ESM.pdf]

Supplementary information

Mesoscopic and microscopic imaging of sensory responses in the same animal

Boido et al. 2019

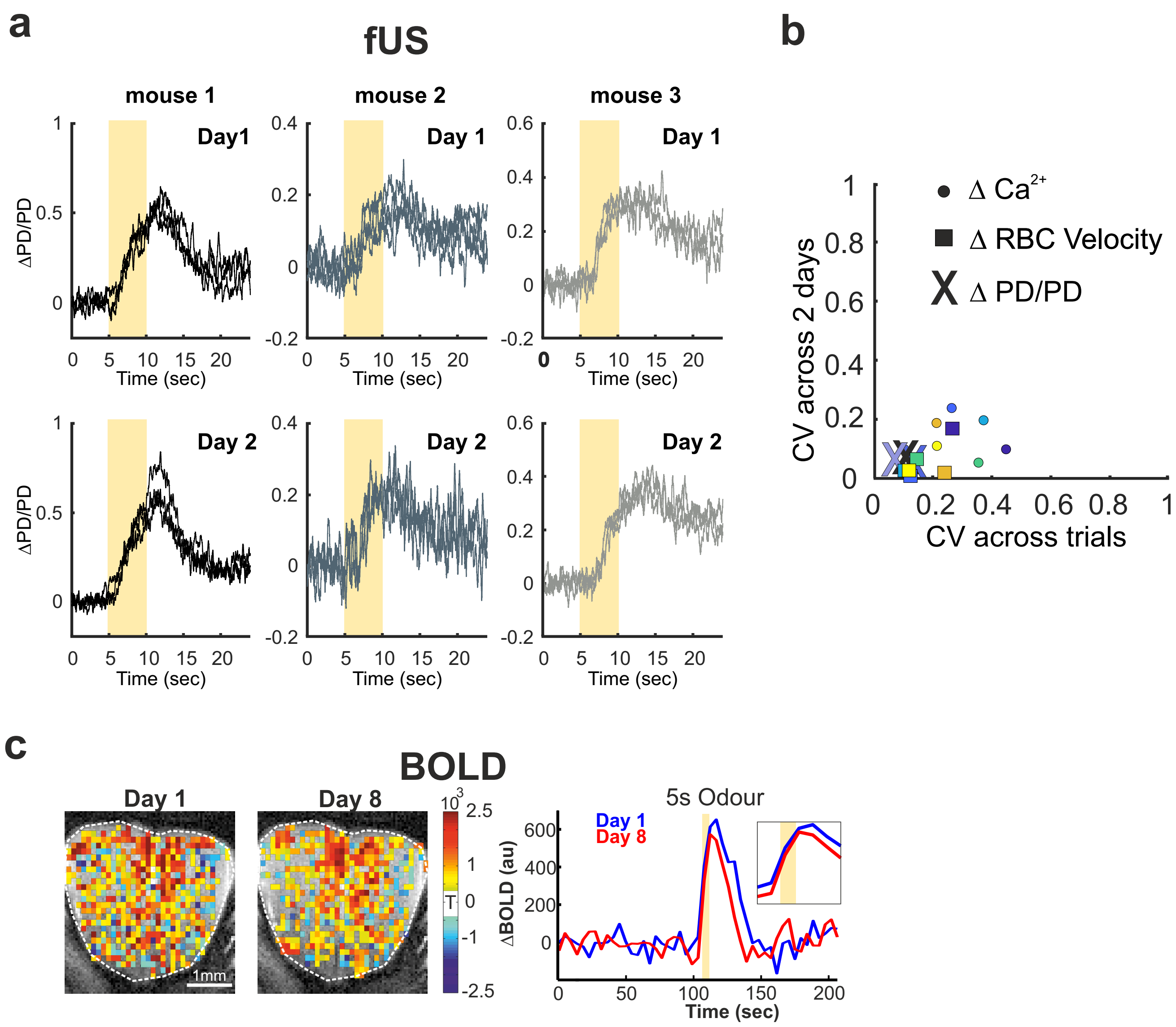

Supplementary Figure 1. Reproducibility of microscopic and mesoscopic responses to odour. (**a**) 3 mice were imaged 3-4 times during two fUS imaging sessions separated by more than 3 weeks, respectively. ΔPD/PD responses to odour are superimposed. Note the highly response reliability on all recording days. (**b**) Quantification of calcium, RBC velocity and ΔPD/PD response reproducibility. The variance across trials is larger than across days for the 3 types of responses. Note that the larger calcium response variability results from the impossibility to draw linescans exactly at the same site (the same postsynaptic dendrites) surrounding a given capillary whereas RBC velocity measurements from the same capillary were absolute measurements (6 capillaries, 3 mice). (**c**) A mouse was imaged during two fMRI imaging sessions separated by 7 days. Left, ΔBOLD activation maps to a 5s inhalation acquired on day 1 and day 8 (averages of 3 inhalations). A color threshold (T in the color bar) was applied to the activation map (for graphical illustration only). Olfactory bulb boundaries (dotted line) when superimposed on an anatomical image acquired using a RARE sequence. Right, response time courses extracted from the entire OB ROI. Note the near perfect overlay of day 1 and day 8 responses. Inset shows an enlargement of the responses.

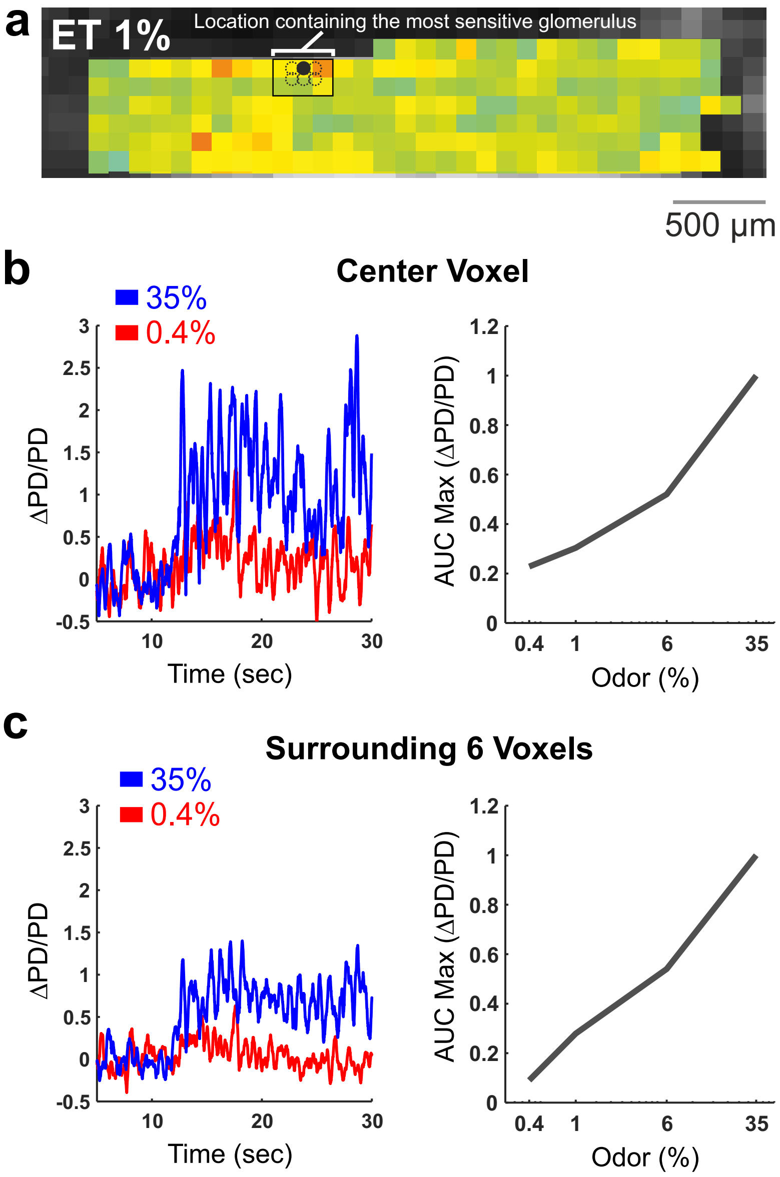

Supplementary Figure 2. Analysis of fUS signals from the region surrounding the most sensitive glomerulus.

(a) ΔPD Map of response to odour at 1% ET with high pass filter of >2.5mm/s as shown in **Figure 4**. Solid black circle indicates the estimated voxel where the most sensitive glomerulus was selected with 2PLSM and analyzed in **(b)**. Dashed line open circles indicate potential locations of the selected glomerulus taking into account the sources of error associated with estimating precise voxel location (see methods). Black rectangle indicates group of six voxels containing the most sensitive glomerulus and analyzed in **(c)**. **(b-c)** Left, time course of ΔPD/PD response to low (0.4%) and high (35%) ET odour (average of 3 inhalations). Right, Semi-log plot of ΔPD/PD AUC maxima vs. odour concentration for the center voxel **(b)** and group of 6 voxels **(c)** (average of 3 inhalations, normalized to 35% ET).

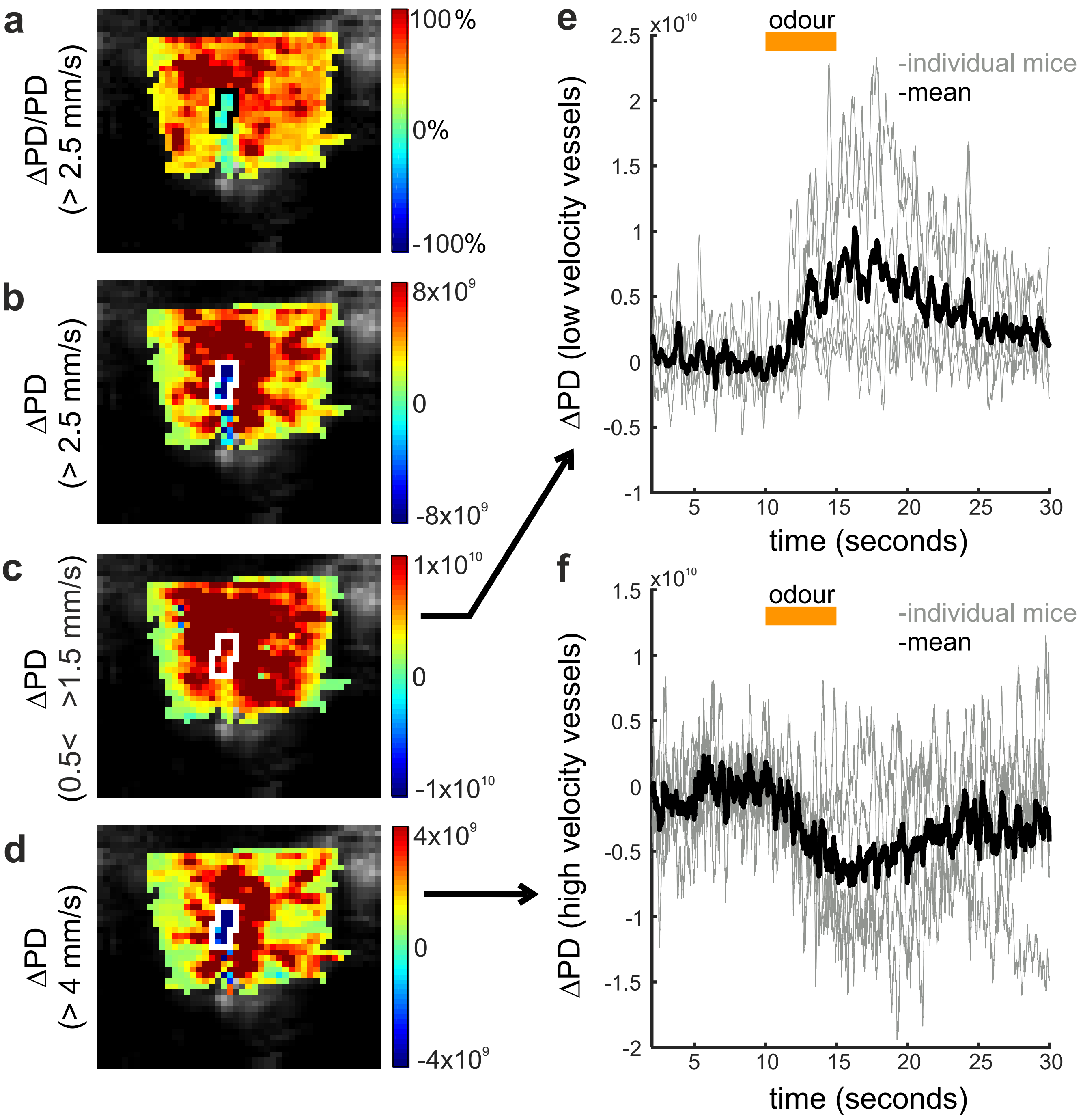

Supplementary Figure 3. Negative responses observed with fUS at high odour concentration in high velocity vessels.

(**a-d**) Maps of fUS responses to 35% ET, with different analysis conditions. ROI indicates region of negative response **(a)** ΔPD/PD response show very minor negative response (green voxels) in comparison to positive responding voxels using an axial velocity high pass filter of > 2.5 mm/s. Same as in Figure 4. **(b)** When shown on a ΔPD map negative voxels become more apparent suggesting that although the net change is large, the negativity is hidden in ΔPD/PD maps because it is located in voxels with high resting PD signal. **(c-f)** When the analysis is selectively filtered for slow velocity (0.5< >1.5 mm/s) vs high velocity (>4 mm/s), negativity is only observed in the high velocity component (d,f), whereas the low velocity filtering show a positive response (c,e). (e,f) Grey traces show analysis of negative responses in 5 mice, whereas black trace shows the mean response.

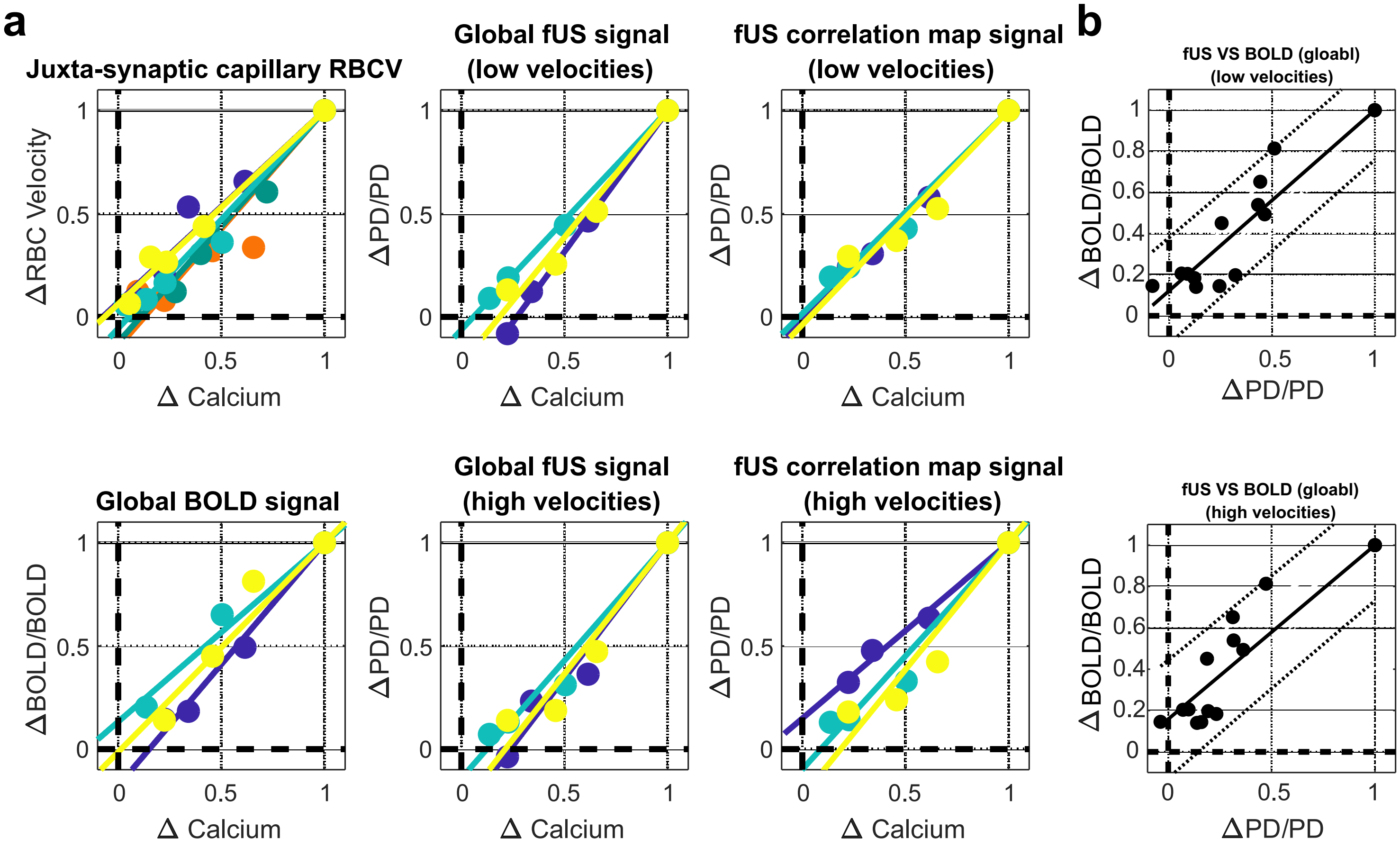

Supplementary Figure 4. Relationships of vascular signals analyzed within individual mice.

(**a**) Same data as Figure 7, except data from individual mice with each technique is fit. Integrals (AUC) of the signals are plotted on both axes, normalized to the response at the highest odour concentration (35% ET). Each colour represents an individual mouse. 5 mice were imaged with TPLSM (calcium and RBC velocity), whereas 3 mice where imaged sequentially across all techniques. Each colour corresponds to an individual mouse. (**b**) Global ΔBOLD/BOLD responses vs. Global ΔfUS/fUS signal (measured within the mask of the entire OB slice). Solid lines show linear fit, dashed lines indicate 95% confidence level of the fit (n = 5 mice).
