# Supplementary material for: Mesoscopic and microscopic imaging of sensory responses in the same animal
Source: Nat Commun. 2019 Mar 7;10:1110. doi: 10.1038/s41467-019-09082-4 (PMC6405955; doi:10.1038/s41467-019-09082-4)
Supplement: Supplementary file 2 — Reporting Summary [file 41467_2019_9082_MOESM2_ESM.pdf]

nature research

Corresponding author(s): Serge Charpak

Last updated by author(s): Feb 15, 2019

Reporting Summary

Nature Research wishes to improve the reproducibility of the work that we publish. This form provides structure for consistency and transparency in reporting. For further information on Nature Research policies, see Authors & Referees and the Editorial Policy Checklist.

Statistics

For all statistical analyses, confirm that the following items are present in the figure legend, table legend, main text, or Methods section.

n/a	Confirmed	
☐	☒	The exact sample size (n) for each experimental group/condition, given as a discrete number and unit of measurement
☐	☒	A statement on whether measurements were taken from distinct samples or whether the same sample was measured repeatedly
☐	☒	The statistical test(s) used AND whether they are one- or two-sided<br>*Only common tests should be described solely by name; describe more complex techniques in the Methods section.*
☐	☒	A description of all covariates tested
☐	☒	A description of any assumptions or corrections, such as tests of normality and adjustment for multiple comparisons
☐	☒	A full description of the statistical parameters including central tendency (e.g. means) or other basic estimates (e.g. regression coefficient) AND variation (e.g. standard deviation) or associated estimates of uncertainty (e.g. confidence intervals)
☐	☒	For null hypothesis testing, the test statistic (e.g. F, t, r) with confidence intervals, effect sizes, degrees of freedom and P value noted<br>*Give P values as exact values whenever suitable.*
☒	☐	For Bayesian analysis, information on the choice of priors and Markov chain Monte Carlo settings
☒	☐	For hierarchical and complex designs, identification of the appropriate level for tests and full reporting of outcomes
☐	☒	Estimates of effect sizes (e.g. Cohen's d, Pearson's r), indicating how they were calculated

Our web collection on statistics for biologists contains articles on many of the points above.

Software and code

Policy information about availability of computer code

Data collection	For TPLSM data we used custom made Labview and Matlab software. For fUS data we used custom made Matlab software. For BOLD fMRI we used Bruker Paravision 5.1.
Data analysis	For all the data we used Labview and Matlab custom made software. For BOLD fMRI we used SPM 12 from Wellcome Trust Centre for Neuroimaging.

For manuscripts utilizing custom algorithms or software that are central to the research but not yet described in published literature, software must be made available to editors/reviewers. We strongly encourage code deposition in a community repository (e.g. GitHub). See the Nature Research guidelines for submitting code & software for further information.

Data

Policy information about availability of data

All manuscripts must include a data availability statement. This statement should provide the following information, where applicable:

- Accession codes, unique identifiers, or web links for publicly available datasets
- A list of figures that have associated raw data
- A description of any restrictions on data availability

Software and date used for this study are available upon reasonable request to the authors.

Field-specific reporting

Please select the one below that is the best fit for your research. If you are not sure, read the appropriate sections before making your selection.

☒ Life sciences ☐ Behavioural & social sciences ☐ Ecological, evolutionary & environmental sciences

October 2018

Life sciences study design

All studies must disclose on these points even when the disclosure is negative.

Sample size	Sample sizes were chose to be consistent with previous studies. Sample size was computed only for minimal stimulation experiments because of the different SNR of the compared signals (intracellular calcium VS capillary RBC velocity)
Data exclusions	No data excluded
Replication	All the results have been showed to be reproducible across time and animals.
Randomization	Randomization was not relevant for this study
Blinding	Although no blind analysis was performed, all the data analyses were accomplished by using automated algorithms in which the arbitrary choices were minimized as much as possible.

Reporting for specific materials, systems and methods

We require information from authors about some types of materials, experimental systems and methods used in many studies. Here, indicate whether each material, system or method listed is relevant to your study. If you are not sure if a list item applies to your research, read the appropriate section before selecting a response.

Materials & experimental systems

n/a	Involved in the study	
☒	☐	Antibodies
☒	☐	Eukaryotic cell lines
☒	☐	Palaeontology
☐	☒	Animals and other organisms
☒	☐	Human research participants
☒	☐	Clinical data

Methods

n/a	Involved in the study	
☒	☐	ChIP-seq
☒	☐	Flow cytometry
☐	☒	MRI-based neuroimaging

Animals and other organisms

Policy information about studies involving animals; ARRIVE guidelines recommended for reporting animal research

Laboratory animals	Thy1-GCaMP6f (GP5.11) mice were obtained from Jackson laboratory. Adult mice (n=13, 2–12 months old, 20–35 g, both males and female, housed in 12-h light-dark cycle, fed ad libitum).
Wild animals	The study did not involve wild animals
Field-collected samples	The study did not involve samples collected from the field
Ethics oversight	All animal care and experimentation was performed in accordance with the INSERM Animal Care and Use Committee guidelines (protocol numbers CEEA34.SC.122.12 and CEEA34.SC.123.12)

Note that full information on the approval of the study protocol must also be provided in the manuscript.

Magnetic resonance imaging

Experimental design

Design type	Mixed design
Design specifications	Single trials lasted 4' (2 minutes baseline, 5 sec odour puff and recovery form activation) with an inter-trial time of at least 1 minute to prevent adaptation to odour. The average experimental session was ~ 2 hours. During this time we managed to record, after field inhomogeneities correction, 4 different odour concentration, 3 trials each.
Behavioral performance measures	Mice were passively sniffing odor puffs delivered during fMRI scans. No behavioral performance requested.

Acquisition

Imaging type(s)	BOLD fMRI
Field strength	17.2T
Sequence & imaging parameters	2D FLASH sequence with the following acquisition parameters: flip angle = 30°, field of view = 0.84 ×0.84 cm2, in plane resolution = 110 × 130 µm2, number of slices = 3, slice thickness = 500 µm, echo time = 6 milliseconds, repetition time = 70 milliseconds, number of repetitions = 50, acquisition time = 3 minutes and 44 seconds. Frames were acquired every 4.48 seconds.
Area of acquisition	Olfactory Bulb

Diffusion MRI ☐ Used ☒ Not used

Preprocessing

Preprocessing software	No preprocessing performed apart from motion correction realignment (no smoothing or segmentation).
Normalization	No data normalization performed. Comparison of activation maps across mice was not the aim of this study.
Normalization template	N/A
Noise and artifact removal	Frames were corrected for movement (slow drift) using motion correction algorithm provided by SPM12.
Volume censoring	No volume censoring applied

Statistical modeling & inference

Model type and settings	Univariate analysis (GLM and T-test). First level analysis only.
Effect(s) tested	We asked whether there was a statistically significant activation on each voxel upon odor application with respect to baseline.

Specify type of analysis: ☐ Whole brain ☒ ROI-based ☐ Both

Anatomical location(s): Olfactory Bulb anatomically located before fMRI sequences.

Statistic type for inference (See Eklund et al. 2016)	Voxel-wise
Correction	No correction for multiple comparisons applied

Models & analysis

n/a	Involved in the study
☒	☐ Functional and/or effective connectivity
☒	☐ Graph analysis
☒	☐ Multivariate modeling or predictive analysis
